# Supplementary material for: Endosomal Arl4A attenuates EGFR degradation by binding to the ESCRT-II component VPS36
Source: Nat Commun. 2023 Nov 29;14:7859. doi: 10.1038/s41467-023-42979-9 (PMC10687025; doi:10.1038/s41467-023-42979-9)
Supplement: Supplementary file 1 — Supplementary Information [file 41467_2023_42979_MOESM1_ESM.pdf]

Supplementary Information

Endosomal Arl4A attenuates EGFR degradation by binding to the ESCRT-II component VPS36

Shin-Jin Lin[1, 2], Ming-Chieh Lin[1, 2], Tsai-Jung Liu[1, 2], Yueh-Tso Tsai[1], Ming-Ting Tsai[1] and Fang-Jen S. Lee[1, 2, 3*]

Supplementary figure 1

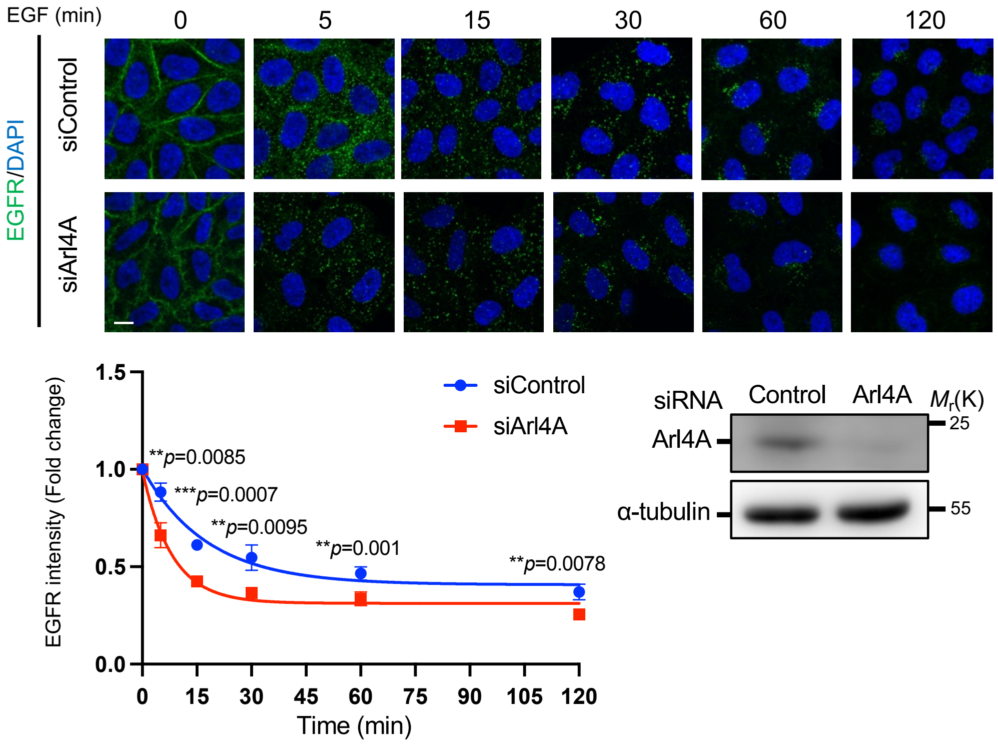

Figure S1. Depletion of Arl4A alters EGFR trafficking. Serum-starved HeLa cells were treated with EGF (80 ng/ml) and EGFR antibody (18 µg/ml) (anti-extracellular region of EGFR, #ab30), fixed at the indicated times, and stained with anti-mouse Alexa-488 secondary antibody and DAPI (blue). The EGFR intensity was determined using a nonlinear one-phase decay fit of the time course. The results represent the mean fluorescence intensity ± SD of three independent experiments and the *p*-values were assessed by two-sided t-test. (each experiment consisted of an average of 30 or more cells). Arl4A knockdown efficiency was assessed by western blotting.

Supplementary figure 2

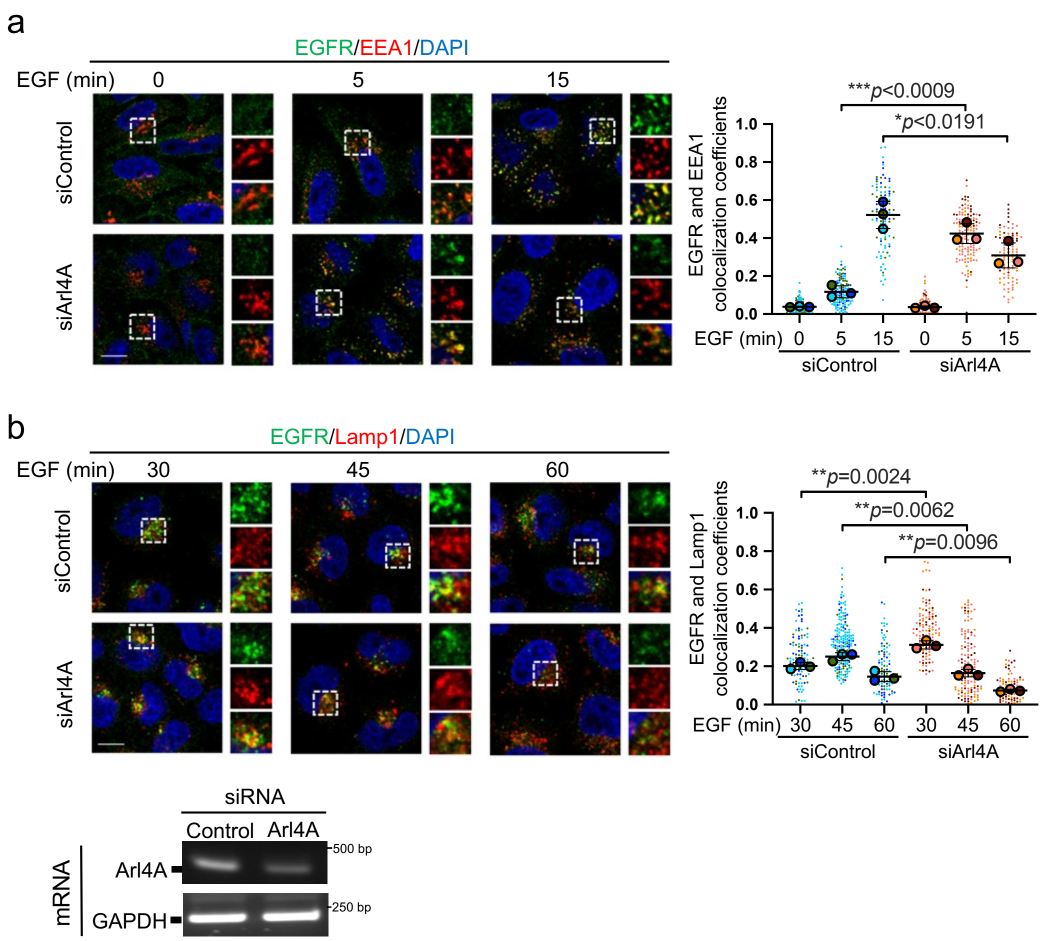

Figure S2. Effects of Arl4A knockdown on EGFR transport in HeLa cells. HeLa cells were transfected for 48 h with siControl or siArl4A. Cells were sequentially incubated at 37°C for 6 h in serum-free medium and finally treated for the indicated time with 100 ng/ml EGF. (a) Confocal sections of HeLa cells stained for EGFR (green), EEA1 (red), and DAPI (blue). Scale bar, 10 μm. Quantification of EGFR and EEA1 colocalization is shown as the colocalization coefficient. The results represent the mean ± SD of three independent experiments and the *p*-values were assessed by two-sided t-test (each experiment consisted of an average of 28 or more cells). (b) Confocal sections of HeLa cells stained for EGFR (green), Lamp1 (red), and DAPI (blue). Scale bar, 10 μm. Quantification of EGFR and Lamp1 colocalization is shown as the colocalization coefficient. The results represent the mean ± SD of three independent experiments and the *p*-values were assessed by two-sided t-test (each experiment consisted of an average of 28 or more cells). Arl4A knockdown efficiency was assessed by RT-PCR. GAPDH was used as the internal control.

Supplementary figure 3

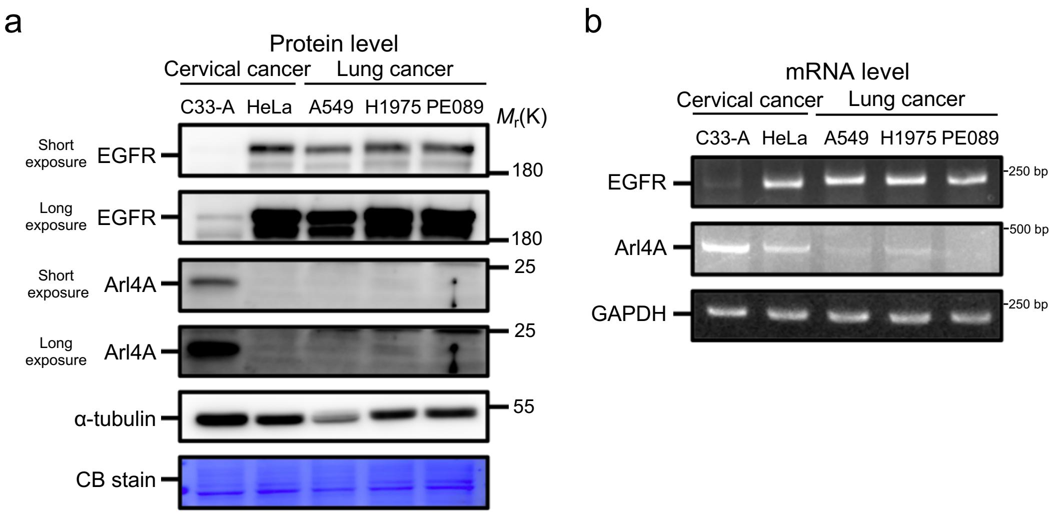

Figure S3. EGFR and Arl4A mRNA and protein expression levels in different cell lines. (a) Cell lysates from different cell lines as indicated were analyzed by immunoblotting with anti-Arl4A, anti-EGFR, and anti-α-tubulin antibodies. (b) mRNA was prepared from different cell lines as indicated. EGFR, Arl4A, and GAPDH mRNA levels were measured by RT-PCR.

Supplementary figure 4

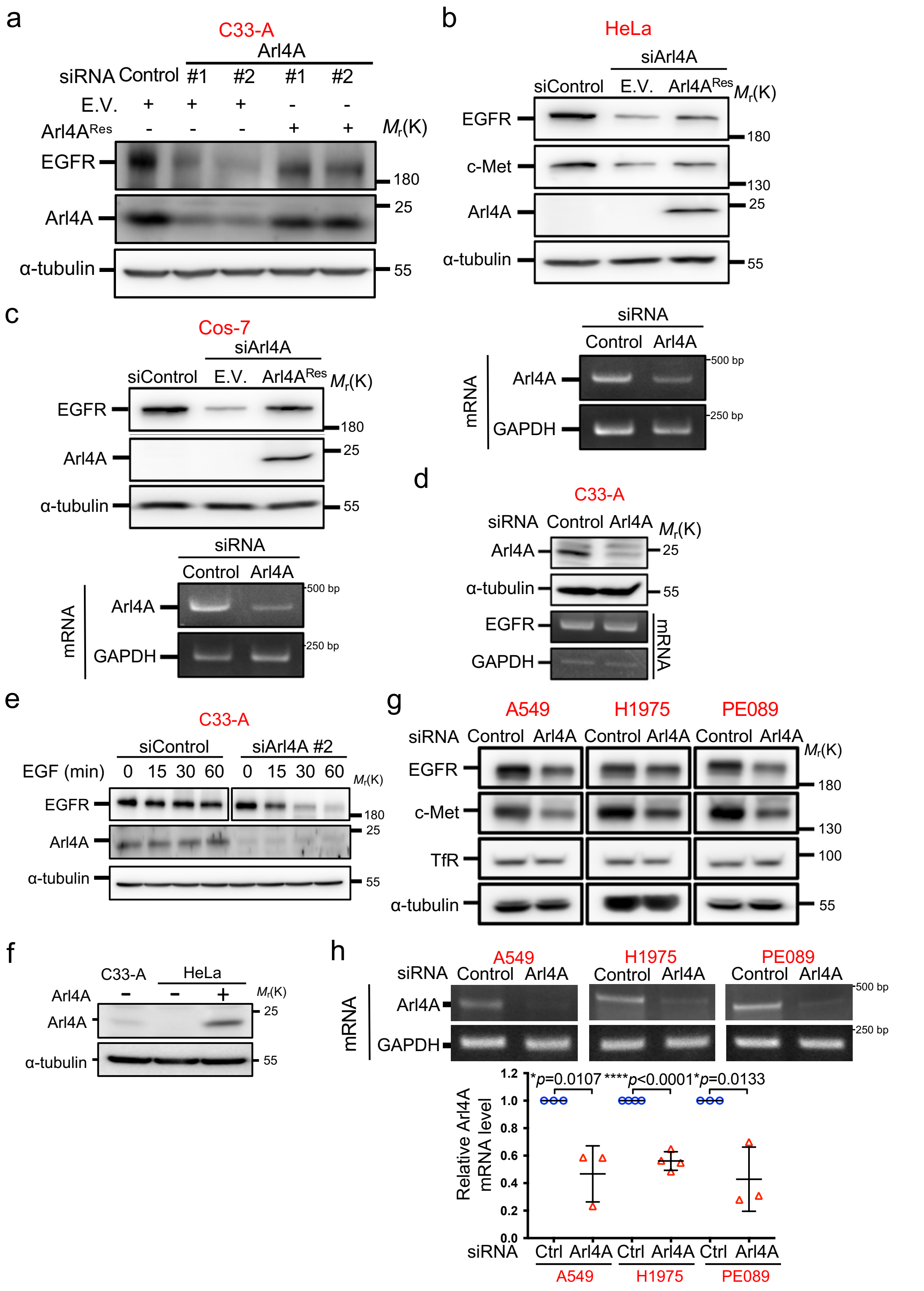

Figure S4. Arl4A regulates RTK protein levels in different cell lines. (a) The effects of Arl4A knockdown by two single siRNAs on EGFR protein levels were rescued by the expression of an Arl4A WT-resistant clone (Arl4A WT^{Res}) in C33-A cells (b-c) The effects of Arl4A knockdown on EGFR protein levels were rescued by the expression of

an Arl4A WT-resistant clone (Arl4A WT^{Res}) in HeLa cells (b) and Cos-7 cells (c) Arl4A$^{WT(Res)}$ contains silent mutations in the RNAi targeting sequence. Cells were treated with either the control or Arl4A siRNA as well as the indicated plasmids. Cell lysates were analyzed by immunoblotting with anti-EGFR, anti-c-Met and anti-α-tubulin antibodies. Arl4A knockdown efficiency was assessed by RT-PCR amplification using Arl4A-specific primers, and GAPDH was used as an internal control (lower panel). (d) Arl4A knockdown did not reduce the EGFR mRNA level. C33-A cells were treated with either the control or Arl4A siRNA. The EGFR mRNA level was measured by RT-PCR. (e) Knockdown and expression of Arl4A regulated the half-life of EGFR degradation upon EGF treatment. Arl4A was knocked down by siArl4A #2 in C33A cells. To compare EGFR degradation rates, we adjusted EGFR protein levels to the same value at time 0 in each group for western blot analysis. (f) The relative amounts of Arl4A in C33-A cells and in HeLa cells after overexpression of Arl4A. HeLa cells were transfected with 1 μg Arl4A plasmid/well in 6-well plates for 24 hours. Cell lysates (~20 μg) were analyzed by immunoblotting with anti-Arl4A and anti-α-tubulin antibodies. (g) A549, H1975, and PE089 cells were treated with either the control or Arl4A siRNA (sample same as Fig. 2a). Cell lysates were analyzed by immunoblotting with anti-EGFR, anti-c-Met, anti-TfR, and anti-α-tubulin antibodies. (h) The knockdown efficiency of Arl4A for Figure 2B-D. Knockdown efficiency of Arl4A was assessed by RT-PCR amplification using Arl4A-specific primers. GAPDH was used as an internal control. Results represent the mean ± SD of three to four independent experiments and the *p*-values were assessed by two-sided t-test.

Supplementary figure 5

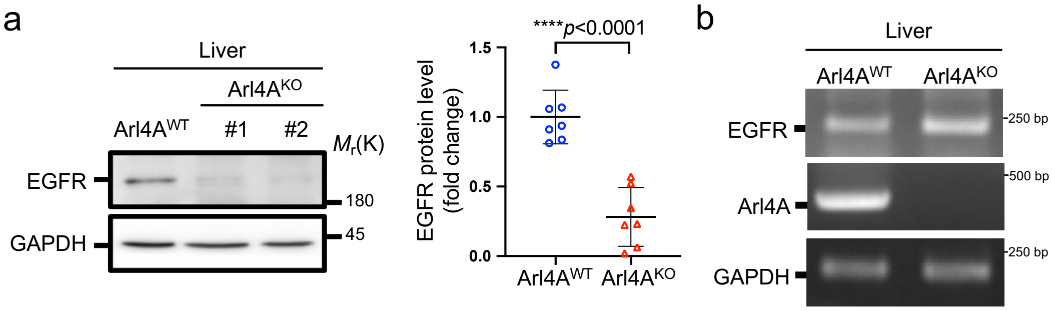

Figure S5. The EGFR protein level is reduced in the Alr4A gene-disrupted mouse livers. (a) We analyzed the expression of EGFR in Arl4A-knockout mouse liver tissue by immunoblotting. Western blotting was performed on whole liver lysates with GAPDH as a loading control (n = 7 mice for whole livers). Quantification of EGFR levels using ImageJ software was shown in the right panel and the *p*-value was assessed by two-sided t-test (n = 7 mice for whole livers). (b) Arl4A knockout did not reduce EGFR mRNA levels. Arl4A and EGFR mRNA levels were measured by RT-PCR.

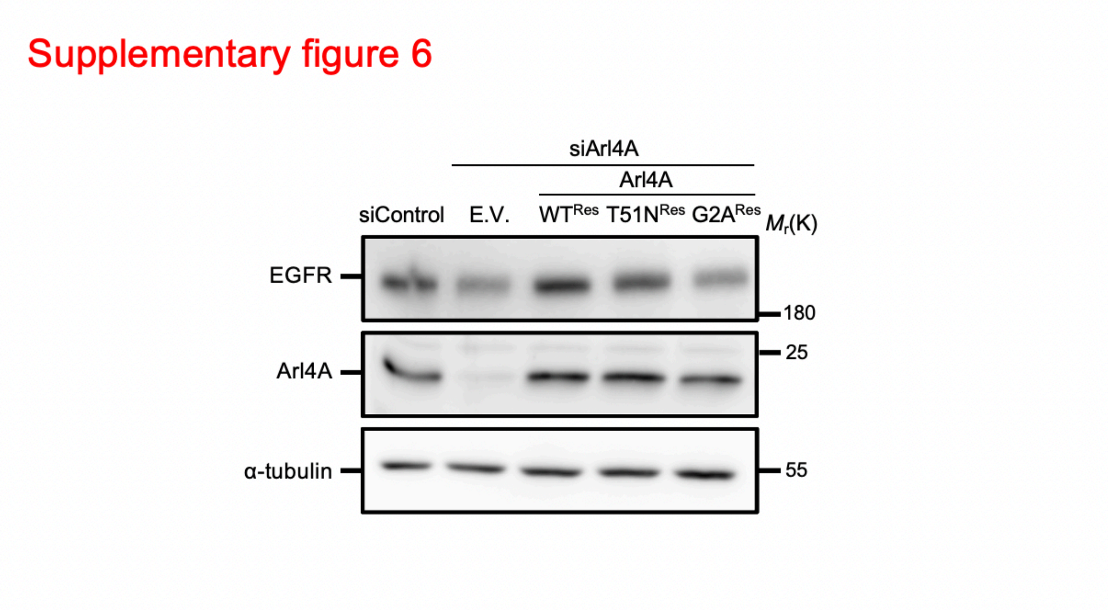

Figure S6. The membrane-targeting ability, but not the nucleotide-binding status, of Arl4A regulates EGFR protein levels. The effects of Arl4A knockdown on the EGFR protein level were rescued by overexpression of Arl4A WT^{Res}, Arl4A T51N^{Res}, or Arl4A G2A^{Res} in C33-A cells. Cells were treated with either the control or Arl4A siRNA as well as the indicated plasmids. Cell lysates were analyzed by immunoblotting with anti-Arl4A, anti-EGFR and anti-α-tubulin antibodies.

Supplementary figure 7

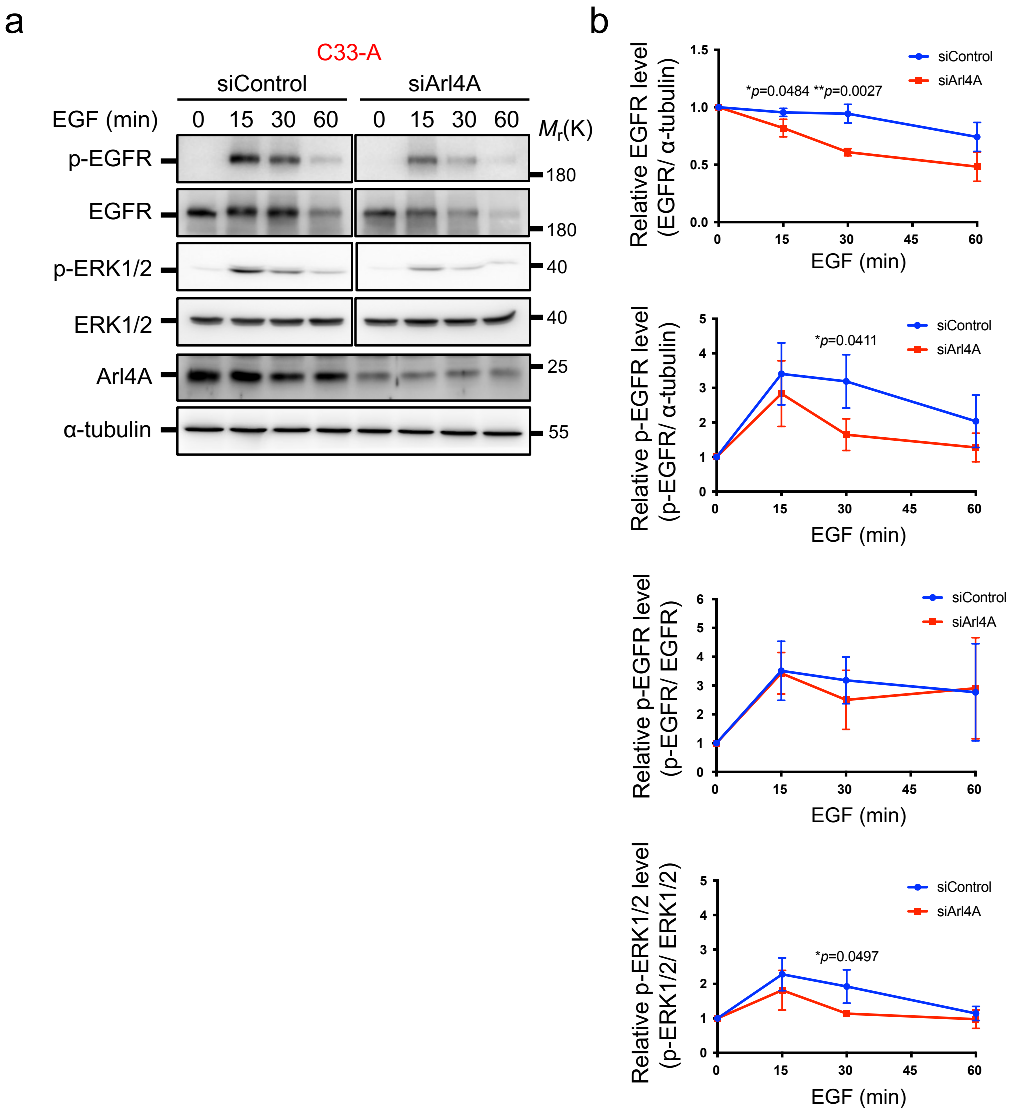

Figure S7. Arl4A depletion negatively modulates EGFR-mediated ERK activation. (a) Cells as indicated were transfected with either the control or Arl4A siRNA. After 48 h of transfection, the cells were starved and stimulated with EGF (100 ng/ml) for the indicated times. Cell lysates were analyzed by immunoblotting with anti-Arl4A, anti-EGFR, anti-p-EGFR, anti-p-ERK1/2, anti-total-ERK1/2 and anti-α-tubulin antibodies. (b) Quantification of p-EGFR, EGFR, and p-ERK1/2 levels were performed using ImageJ software. The p-EGFR and EGFR expression levels were normalized for equal loading as determined with α-tubulin. The relative phosphorylation levels of EGFR and ERK1/2 were normalized to their total non-phosphorylated forms at corresponding time points. Results represent the mean ± SD of three independent experiments and the *p*-values were assessed by two-sided t-test.

Supplementary figure 8

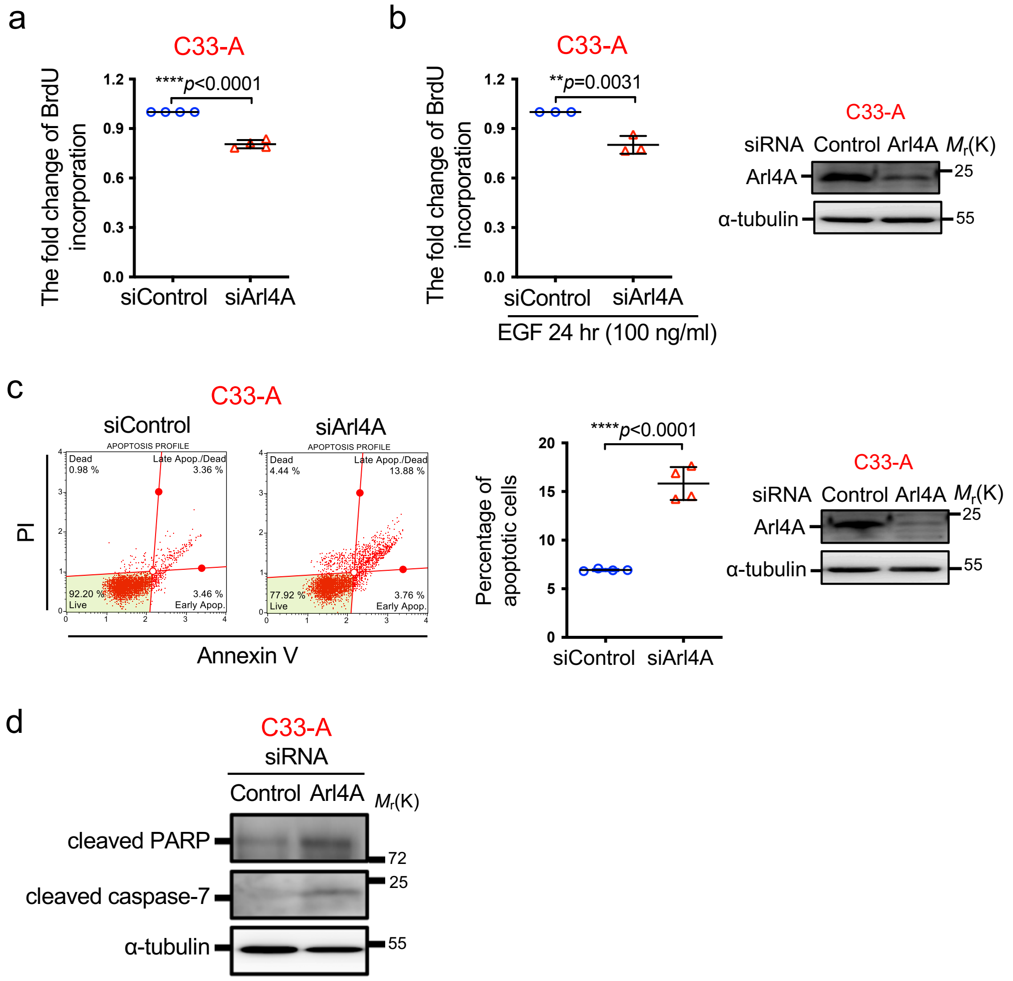

Figure S8. Depletion of Arl4A inhibits cell proliferation and induces apoptosis. (a) C33-A cells were transfected with either the control or Arl4A siRNA. After 48 h of transfection, a BrdU assay was performed to estimate cell proliferative activity. The results represent the mean ± SD of four independent experiments and the *p*-value was assessed by two-sided t-test. (b) C33-A cells were transfected with either the control or Arl4A siRNA. After 24 h, the cells were treated with EGF (100 ng/ml) for 24 h. After 24 h of EGF treatment, a BrdU assay was performed to estimate cell proliferative activity. The results represent the mean ± SD of three independent experiments and the *p*-value was assessed by two-sided t-test. (c) C33-A cells were transfected with either the control or Arl4A siRNA. After 24 h of transfection, apoptotic cells were measured by flow cytometry with PI and annexin V staining. The results represent the mean ± SD of three independent experiments and the *p*-value was assessed by two-sided t-test. (d) Cell lysates were analyzed by immunoblotting with anti-cleaved PARP, anti-cleaved caspase-7 and anti-α-tubulin antibodies. The results shown are representative of at least

three independent experiments.

Supplementary figure 9

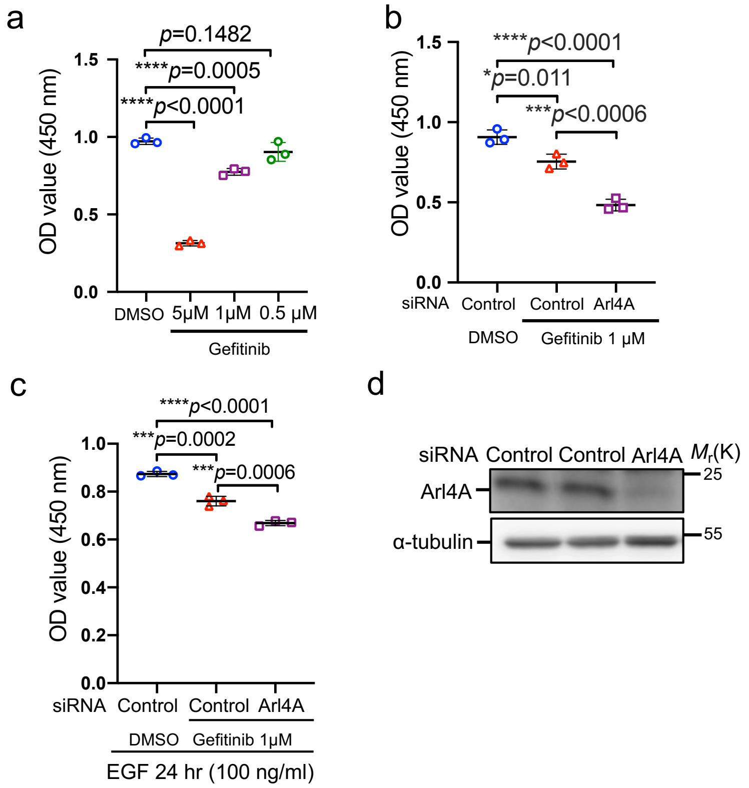

Figure S9. Effect of Arl4 on cell proliferation. (a) C33-A cells treated with different concentrations of gefitinib for 24 h as indicated. (b) C33-A cells were transfected with either control or Arl4A siRNA. After 48 h of transfection, gifitinib (1 μM) was added for 24 hours. (c) C33-A cells were transfected with either control or Arl4A siRNA. After 48 h of transfection, gefitinib (1 μM) and EGF (100 ng/ml) were added for 24 hours. (a-c) To determine cell viability, a WST-1 assay was performed as described in Materials and Methods. Results represent the mean ± SD of three independent experiments and the *p*-values were assessed by one-way ANOVA with Tukey's test. (d) Cell lysates were analyzed by immunoblotting with anti-Arl4A and anti-α-tubulin antibodies.

Supplementary figure 10

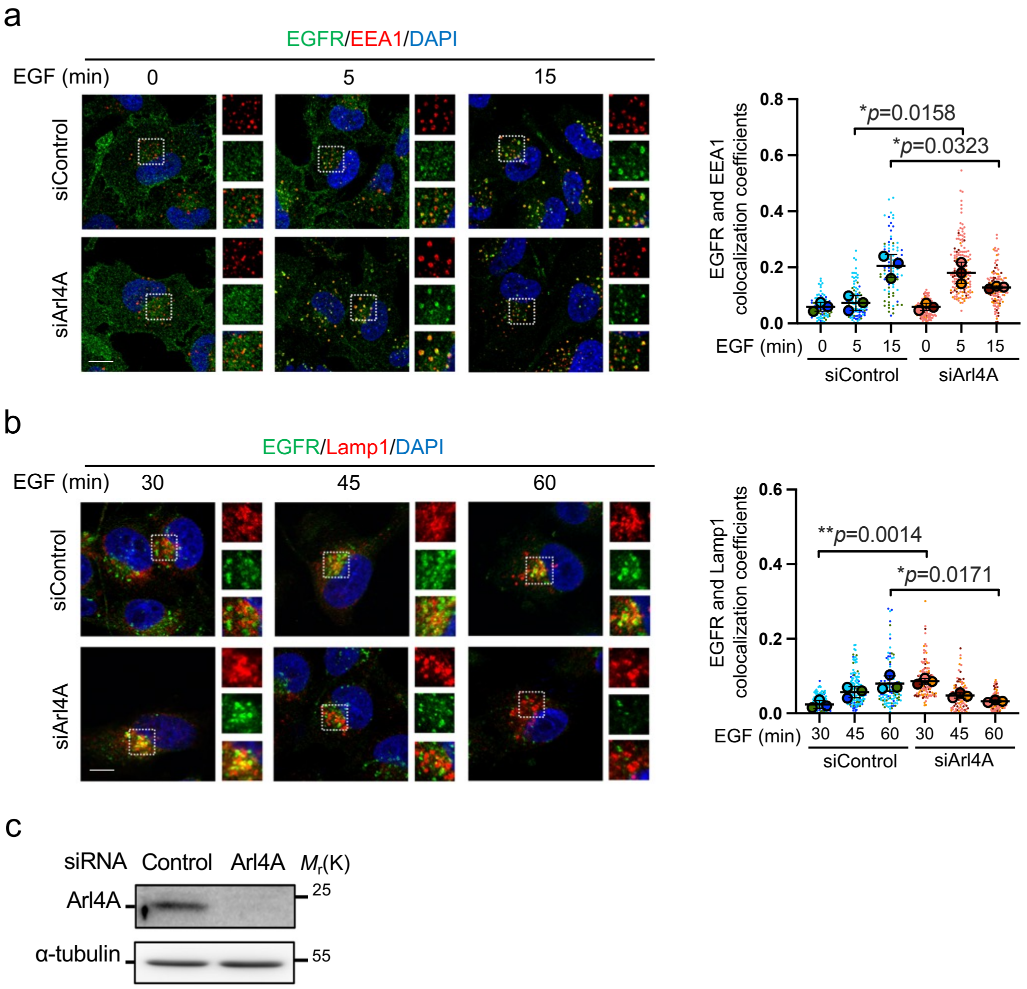

Figure S10. Effects of Arl4A knockdown on EGFR transport in A549 cells. A549 cells were transfected with siControl or siArl4A for 48 h and then serum starved for 6 h before 100 ng/ml EGF treatment for indicated times. (a) Confocal sections of A549 cells stained for EGFR (green), EEA1 (red), and DAPI (blue). Scale bar, 10 µm. Quantification of EGFR and EEA1 colocalization is shown as the colocalization coefficient. The results represent the mean ± SD of three independent experiments and the *p*-values were assessed by two-sided t-test (each experiment consisted of an average of 28 or more cells). (b) Confocal sections of A549 cells stained for EGFR (green), Lamp1 (red), and DAPI (blue). Scale bar, 10 µm. Quantification of EGFR and Lamp1 colocalization is shown as the colocalization coefficient. The results represent the mean ± SD of three independent experiments and the *p*-values were assessed by two-sided t-test (each experiment consisted of an average of 36 or more cells). (c) Arl4A knockdown efficiency in A549 cells. Approximately 120 mg of total protein was analyzed by western blotting with anti-Arl4A and anti-α-tubulin antibodies.

Supplementary figure 11

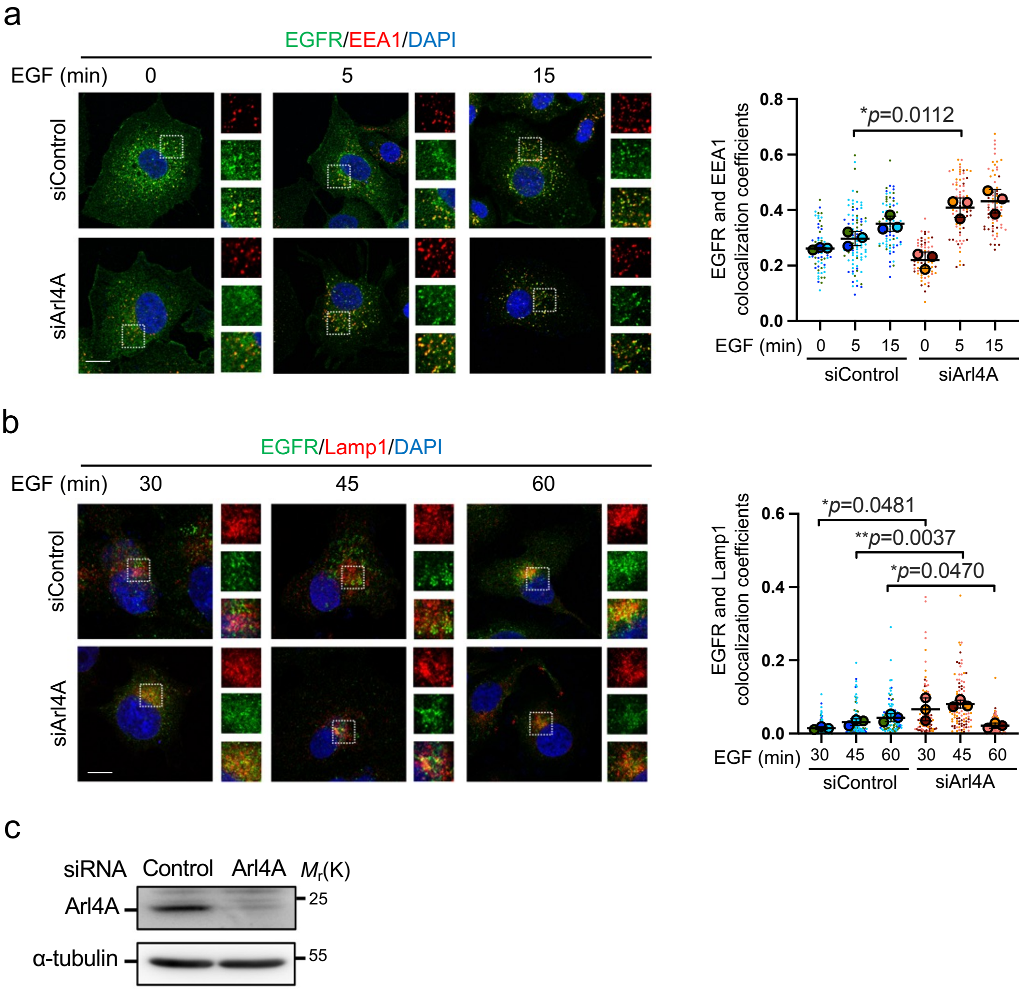

Figure S11. Effects of Arl4A knockdown on EGFR transport in H1975 cells. H1975 cells were transfected with siControl or siArl4A for 48 h and then serum starved for 6 h before 100 ng/ml of EGF treatment for indicated times. (a) Confocal sections of H1975 cells stained for EGFR (green), EEA1 (red), and DAPI (blue). Scale bar, 10 µm. Quantification of EGFR and EEA1 colocalization is shown as the colocalization coefficient. The results represent the mean ± SD of three independent experiments and the *p*-value was assessed by two-sided t-test (each experiment consisted of an average of 22 or more cells). (b) Confocal sections of H1975 cells stained for EGFR (green), Lamp1 (red), and DAPI (blue). Scale bar, 10 µm. Quantification of EGFR and Lamp1 colocalization is shown as the colocalization coefficient. The results represent the mean ± SD of three independent experiments and the *p*-values were assessed by two-sided t-test (each experiment consisted of an average of 30 or more cells). (c) Arl4A knockdown efficiency in H1975 cells. Approximately 120 mg of total protein was analyzed by Western blotting with anti-Arl4A and anti-α-tubulin antibodies.

Supplementary figure 12

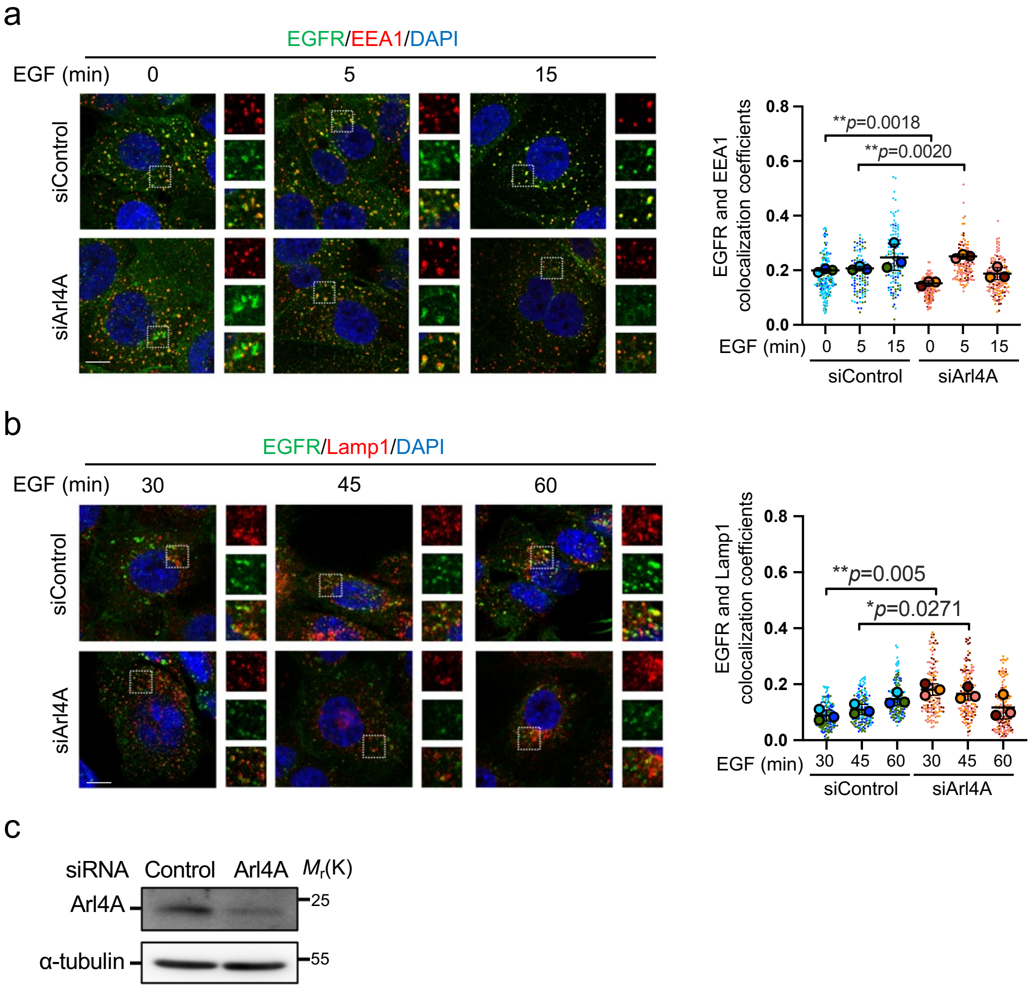

Figure S12. Effects of Arl4A knockdown on EGFR transport in PE089 cells. PE089 cells were transfected with siControl or siArl4A for 48 h and then serum starved for 6 h before 100 ng/ml of EGF treatment for indicated times. (a) Confocal sections of PE089 cells stained for EGFR (green), EEA1 (red), and DAPI (blue). Scale bar, 10 µm. Quantification of EGFR and EEA1 colocalization is shown as the colocalization coefficient. The results represent the mean ± SD of three independent experiments and the *p*-values were assessed by two-sided t-test (each experiment consisted of an average of 35 or more cell). (b) Confocal sections of PE089 cells stained for EGFR (green), Lamp1 (red), and DAPI (blue). Scale bar, 10 µm. Quantification of EGFR and Lamp1 colocalization is shown as the colocalization coefficient. The results represent the mean ± SD of three independent experiments and the *p*-values were assessed by two-sided t-test (each experiment consisted of an average of 40 or more cells). (c) Arl4A knockdown efficiency in PE089 cells. Approximately 120 mg of total protein was analyzed by western blotting with anti-Arl4A and anti-α-tubulin antibodies.

Supplementary figure 13

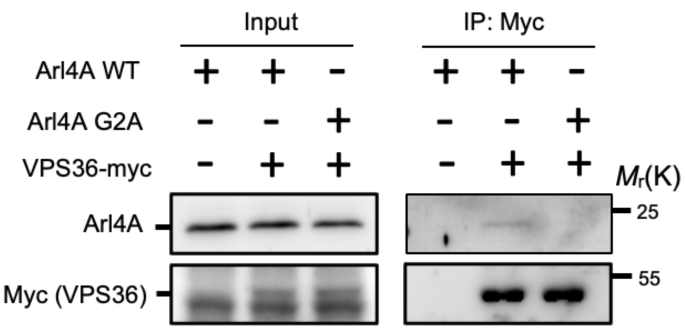

Figure S13. Arl4A G2A mutant does not bind to VPS36. Wild-type Arl4A or the G2A mutant interacted with VPS36-myc *in vivo*. Lysates of HeLa cells transfected with the indicated plasmids were immunoprecipitated with Myc-Trap, and bound proteins were analyzed by western blotting with anti-Myc and anti-Arl4A antibodies.

Supplementary figure 14

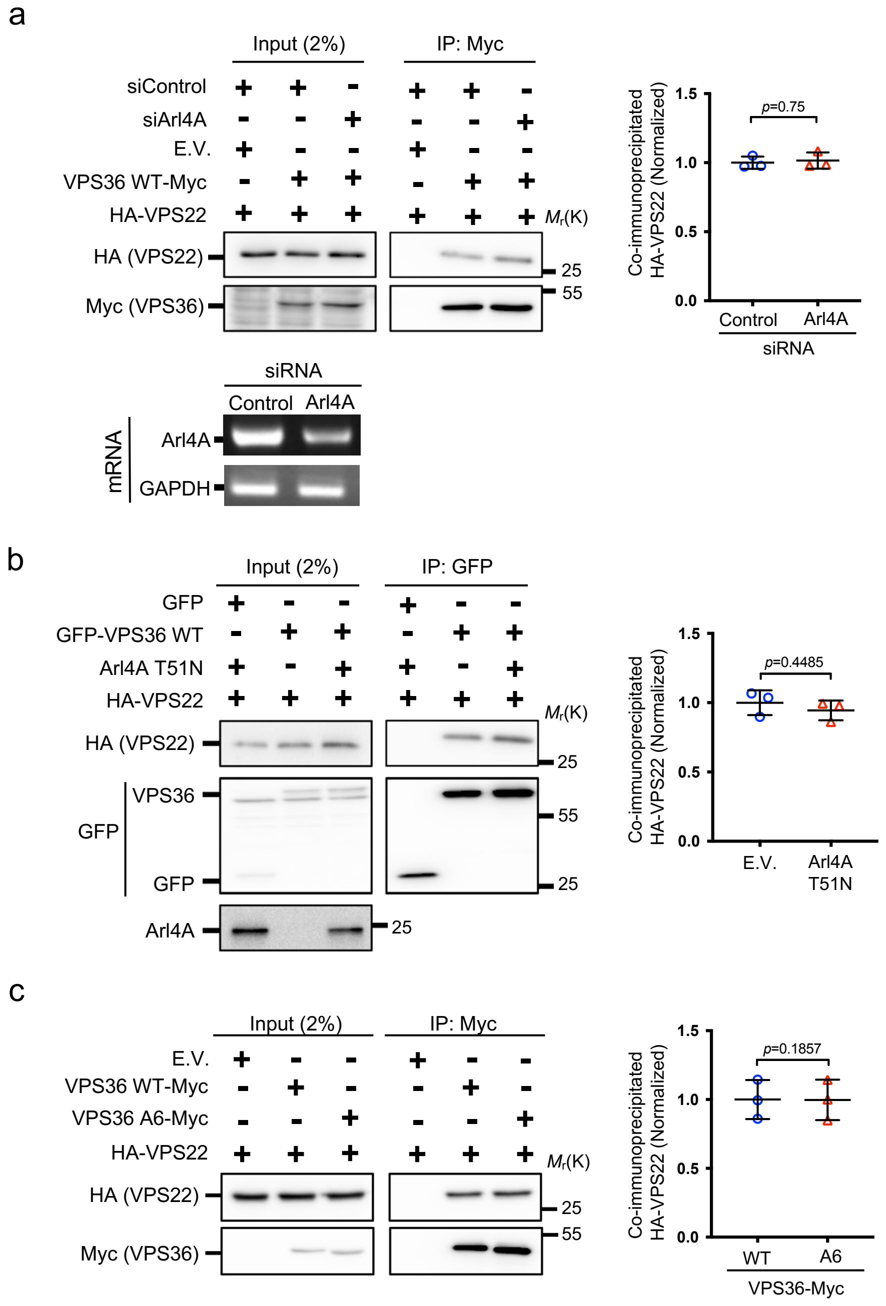

Figure S14. Arl4A binding does not alter the ESCRT-II VPS36-VPS22 interaction. (a) Effects of Arl4A knockdown on the VPS36 interaction with VPS22 in HeLa cells. Lysates of cells transfected with the control or Arl4A siRNA as well as the indicated plasmids were immunoprecipitated with Myc-Trap, and the bound proteins were analyzed by western blotting with the indicated antibodies. The amounts of coimmunoprecipitated VPS22-HA were determined by densitometric quantification from three experiments and the *p*-value was assessed by two-sided t-test. Arl4A knockdown efficiency was assessed by RT-PCR. GAPDH was used as the internal control (lower panel). (b) Effects of Arl4A T51N overexpression on the VPS36

interaction with VPS22 in HeLa cells. Lysates of cells transfected with the indicated plasmids were immunoprecipitated with GFP-Trap, and the bound proteins were analyzed by western blotting with the indicated antibodies. The amounts of coimmunoprecipitated VPS22-HA were determined by densitometric quantification from three experiments and the *p*-value was assessed by two-sided t-test. (c) Comparison of VPS36-VPS22 and A6-VPS22 interactions in HeLa. Cells transfected with HA-VPS22 plus an empty vector (E.V.) or plasmids encoding VPS36 WT-Myc or A6-Myc mutants were immunoprecipitated with Myc-Trap, and immunoprecipitated proteins were analyzed by western blotting with antibodies against Myc and HA. The amounts of coimmunoprecipitated VPS22-HA were determined by densitometric quantification from three experiments. The results represent the mean ± SD of three independent experiments and the *p*-value was assessed by two-sided t-test.

Supplementary figure 15

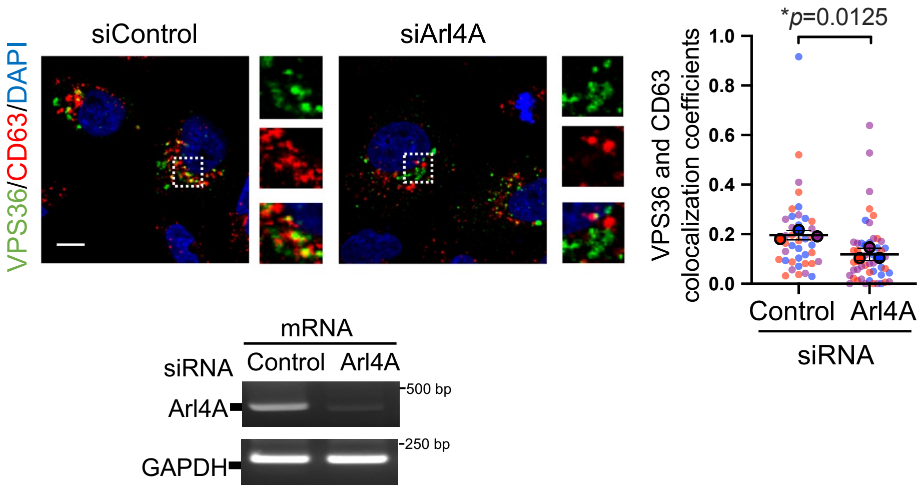

Figure S15. Depletion of Arl4A decreased the ability of VPS36 to localize to MVBs. HeLa cells were transfected with siControl or siArl4A for 48 h and stained with anti-Myc (VPS36-WT-Myc) (green), anti-CD63 (red) and DAPI (blue). Scale bar, 10 µm. Quantification of VPS36 and CD63 colocalization is shown as the Pearson coefficient. Results represent the mean ± SD of three independent experiments and the *p*-value was assessed by two-sided t-test (each experiment consisted of an average of 13 or more cells). Arl4A-knockdown efficiency was assessed by RT-PCR.

Supplementary figure 16

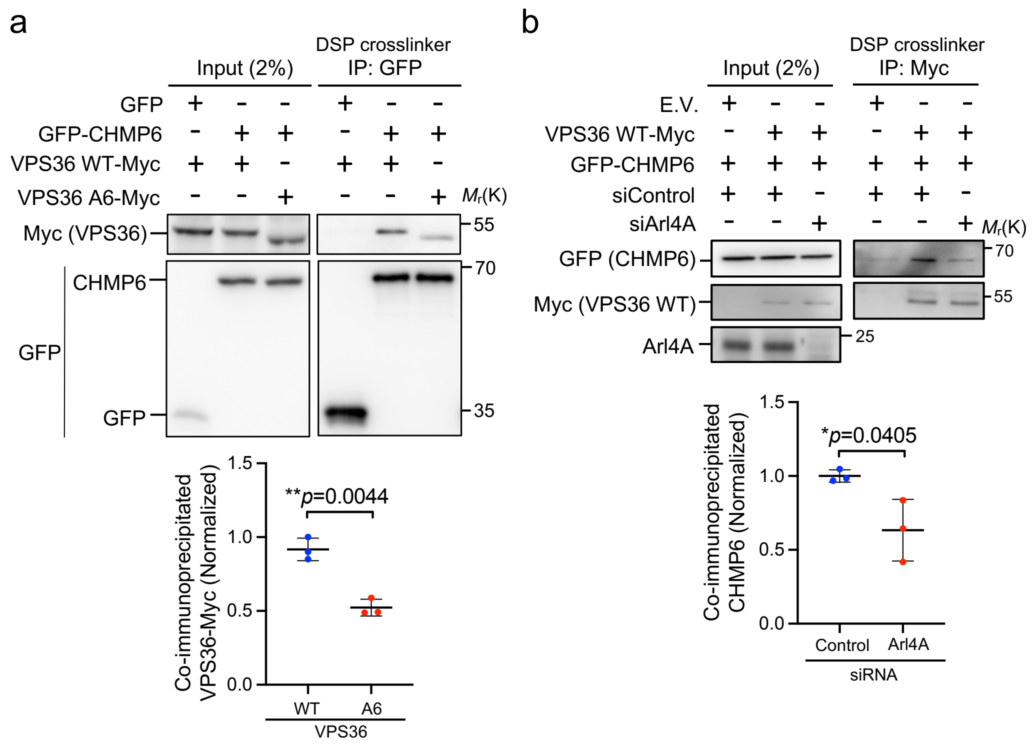

Figure S16 Arl4A affects the association of VPS36 and ESCRT-III CHMP6. (a) Comparison of VPS36-WT and VPS36-A6 binding ability toward ESCRT-III component CHMP6 *in vivo*. HeLa cells transfected with indicated plasmids were treated with 1 mM dithiobis(succinimidyl propionate) (DSP) for 2 hours and quenched by Tris-HCl before cell lysis and immunoprecipitation with GFP-Trap. The bound proteins were analyzed by western blotting with anti-Myc or anti-GFP antibodies. The amounts of coimmunoprecipitated VPS36-Myc were determined by densitometric quantification from three biological experiments and the *p*-value was assessed by two-sided t-test. (b) Co-IP of VPS36 and CHMP6 in Arl4A-depleted cells. C33-A cells transfected with the control or Arl4A siRNA as well as the indicated plasmids were treated with 1 mM DSP for 1 hour and quenched by Tris-HCl before cell lysis and immunoprecipitation with Myc-Trap. The bound proteins were analyzed by western blotting with indicated antibodies. The amounts of coimmunoprecipitated GFP-CHMP6 were determined by densitometric quantification from three biological experiments and the *p*-value was assessed by two-sided t-test.

Supplementary figure 17

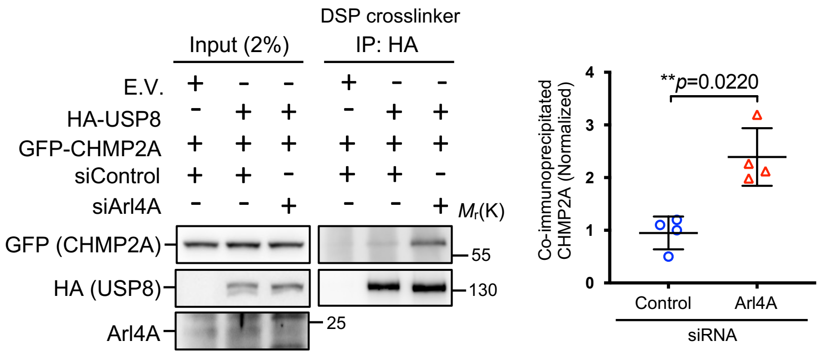

Figure S17 Depletion of Arl4A increases the interaction between CHMP2A and USP8. Co-IP of USP8 and CHMP2A in Arl4A-knockdown HeLa cells. Cells transfected with the control or Arl4A siRNA as well as the indicated plasmids were treated with 1 mM dithiobis(succinimidyl propionate) (DSP) for 2 hours and quenched before cell lysis and immunoprecipitation with HA-beads. The protein inputs and the immunoprecipitates were analyzed by western blotting with the indicated antibodies. The amounts of coimmunoprecipitated GFP-CHMP2A were determined by densitometric quantification from four biological experiments and the *p*-value was assessed by two-sided t-test.

Supplementary figure 18

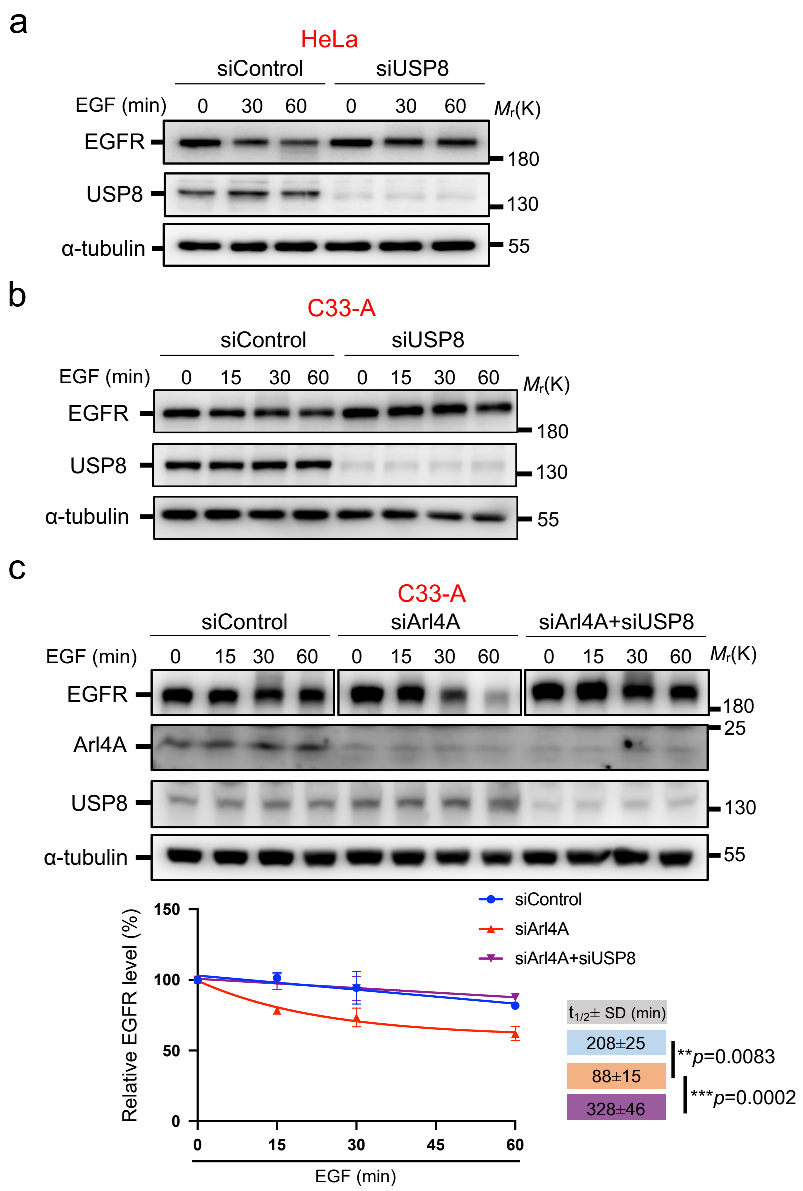

Figure S18. Depletion of USP8 prolongs the half-life of EGFR in Arl4A-depleted cells. (a) HeLa cells and (b) C33-A cells transfected with either the control or USP8 siRNA were subjected to EGFR degradation assays as described in the Materials and Methods. (c) C33-A cells transfected with indicated siRNAs were subjected to EGFR degradation assays as described in the Materials and Methods. To compare EGFR degradation rates, we adjusted EGFR protein levels to the same value at time 0 in each group for western blot analysis. Results represent the mean ± SD of three independent experiments. The t $_{1/2}$ ± SD of EGFR was obtained using a nonlinear one-phase decay fit of the time course and the *p*-values were assessed by one-way ANOVA with Tukey's test.

Supplementary figure 19

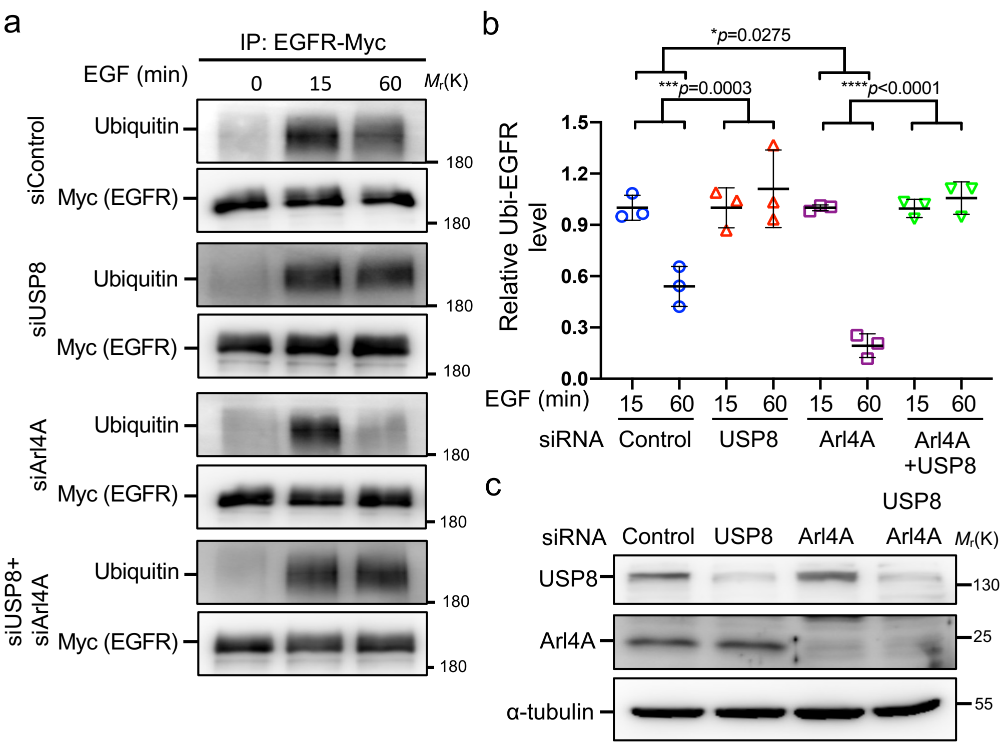

Figure S19. Depletion of USP8 prolongs the duration of EGFR ubiquitination in Arl4A-depleted cells. (a) C33-A cells were transfected with either control, USP8, or Arl4A, or Arl4A combined with USP8 siRNA and EGFR-Myc. After 48 h, cells were starved and stimulated with EGF (100 ng/ml) for the indicated times. Cell lysates were immunoprecipitated with Myc-Trap and analyzed by western blotting with antibodies against ubiquitin. (b) Quantification of ubiquitin-EGFR was performed using ImageJ software. Graph was generated by densitometrically scanning the 60-min ubiquitin-EGFR signal relative to the 15-min ubiquitin-EGFR signal. The relative amount of ubiquitinated EGFR after 60 min was compared by one-way ANOVA. Results represent the mean ± SD of three independent experiments and the *p*-values were assessed by one-way ANOVA with Tukey's test. (c) Lysates of cells used in Figure S19 were analyzed by western blotting with antibodies against USP8, Arl4A, and α-tubulin.
