# Supplementary material for: Endosomal Arl4A attenuates EGFR degradation by binding to the ESCRT-II component VPS36
Source: Nat Commun. 2023 Nov 29;14:7859. doi: 10.1038/s41467-023-42979-9 (PMC10687025; doi:10.1038/s41467-023-42979-9)
Supplement: Supplementary file 3 — Reporting Summary [file 41467_2023_42979_MOESM3_ESM.pdf]

nature portfolio

Corresponding author(s): Fang-Jen S. Lee

Last updated by author(s): Oct 12, 2023

Reporting Summary

Nature Portfolio wishes to improve the reproducibility of the work that we publish. This form provides structure for consistency and transparency in reporting. For further information on Nature Portfolio policies, see our Editorial Policies and the Editorial Policy Checklist.

Statistics

For all statistical analyses, confirm that the following items are present in the figure legend, table legend, main text, or Methods section.

n/a	Confirmed	
[ ]	[x]	The exact sample size (n) for each experimental group/condition, given as a discrete number and unit of measurement
[ ]	[x]	A statement on whether measurements were taken from distinct samples or whether the same sample was measured repeatedly
[ ]	[x]	The statistical test(s) used AND whether they are one- or two-sided<br>*Only common tests should be described solely by name; describe more complex techniques in the Methods section.*
[x]	[ ]	A description of all covariates tested
[x]	[ ]	A description of any assumptions or corrections, such as tests of normality and adjustment for multiple comparisons
[ ]	[x]	A full description of the statistical parameters including central tendency (e.g. means) or other basic estimates (e.g. regression coefficient) AND variation (e.g. standard deviation) or associated estimates of uncertainty (e.g. confidence intervals)
[ ]	[x]	For null hypothesis testing, the test statistic (e.g. F, t, r) with confidence intervals, effect sizes, degrees of freedom and P value noted<br>*Give P values as exact values whenever suitable.*
[x]	[ ]	For Bayesian analysis, information on the choice of priors and Markov chain Monte Carlo settings
[x]	[ ]	For hierarchical and complex designs, identification of the appropriate level for tests and full reporting of outcomes
[ ]	[x]	Estimates of effect sizes (e.g. Cohen's d, Pearson's r), indicating how they were calculated

Our web collection on statistics for biologists contains articles on many of the points above.

Software and code

Policy information about availability of computer code

Data collection	No software used
Data analysis	We used GraphPad Prism8. For western blot images analysis in ImageJ. For microscopy images colocalization analysis in ZEN and Imaris. The propidium iodide (PI) and annexin V-FITC stained cells were analyzed via flow cytometry with a Muse® Cell Analyzer (luminex)

For manuscripts utilizing custom algorithms or software that are central to the research but not yet described in published literature, software must be made available to editors and reviewers. We strongly encourage code deposition in a community repository (e.g. GitHub). See the Nature Portfolio guidelines for submitting code & software for further information.

Data

Policy information about availability of data

All manuscripts must include a data availability statement. This statement should provide the following information, where applicable:

- Accession codes, unique identifiers, or web links for publicly available datasets
- A description of any restrictions on data availability
- For clinical datasets or third party data, please ensure that the statement adheres to our policy

There are no restriction for any materials used in this study. All data generated or analyzed during this study are included in this published article and its supplementary information files. Source data are provided with this paper.

Research involving human participants, their data, or biological material

Policy information about studies with human participants or human data. See also policy information about sex, gender (identity/presentation), and sexual orientation and race, ethnicity and racism.

Reporting on sex and gender	No human research participants
Reporting on race, ethnicity, or other socially relevant groupings	No human research participants
Population characteristics	No human research participants
Recruitment	No human research participants
Ethics oversight	No human research participants

Note that full information on the approval of the study protocol must also be provided in the manuscript.

Field-specific reporting

Please select the one below that is the best fit for your research. If you are not sure, read the appropriate sections before making your selection.

- [x] Life sciences
- [ ] Behavioural & social sciences
- [ ] Ecological, evolutionary & environmental sciences

For a reference copy of the document with all sections, see nature.com/documents/nr-reporting-summary-flat.pdf

Life sciences study design

All studies must disclose on these points even when the disclosure is negative.

Sample size	No sample-size calculations were performed. All data collected from at least three biological replicates are presented as the means±SD. Statistical comparisons between treatments were performed by nonparametric t tests (Student's t tests) or one-way analysis of variance (ANOVA) in GraphPad Prism 8. Sample sizes were determined based on our previous experiments to sufficiently detect meaningful biological different with good reproducibility (Li et al.,2007, Lin et al.,2011, Chiang et al., 2019, Chenet al., 2020 cite in the manuscript)
Data exclusions	No data were excluded.
Replication	All experiments were performed at least three times unless otherwise indicated, and the figures show representative results. The results of immunofluorescence image were repeated three independently with similar results, and one representative image from each group was shown in Fig. 1a, 3c, 4d, 5a, 7a-c and 8a. Figures 4a-b and 6a were independently repeated twice with similar results. Figure 9c and 9f were independently repeated three and five times respectively with similar results.
Randomization	No human subjects were used in the study. Randomization is not generally used in this field. Randomization for different experimental groups was not relevant as they were performed on uniform biological material, ie. Cell lines procured from commercial source. Due to the high reproducibility and consistency between cell cultures, it was predetermined that three biological replicates would allow for adequate analysis to reach meaniful conclusions of the data.
Blinding	Blinding is not relevant to our study because it is not clinical trial or research with different groups/participants

Reporting for specific materials, systems and methods

We require information from authors about some types of materials, experimental systems and methods used in many studies. Here, indicate whether each material, system or method listed is relevant to your study. If you are not sure if a list item applies to your research, read the appropriate section before selecting a response.

Materials & experimental systems

n/a	Involved in the study	
[ ]	[x]	Antibodies
[ ]	[x]	Eukaryotic cell lines
[x]	[ ]	Palaeontology and archaeology
[ ]	[x]	Animals and other organisms
[x]	[ ]	Clinical data
[x]	[ ]	Dual use research of concern
[x]	[ ]	Plants

Methods

n/a	Involved in the study	
[x]	[ ]	ChIP-seq
[ ]	[x]	Flow cytometry
[x]	[ ]	MRI-based neuroimaging

Antibodies

Antibodies used

The antibodies used in this study were as follows: anti-VPS36 (#PA5-60561, Invitrogen,1:1000); and anti-HA (# MMS-101R, Biolegend,1:3000); anti-LexA (Covance,1:12000); anti-Myc (#2276S,1:3000), anti-mCherry (#43590,1:3000), anti-EGFR (#4267,1:3000), anti-p-EGFR (#3777,1:2000), anti-ERK1/2 (#9107,1:3000), anti-p-ERK1/2 (4370,1:3000), anti-c-Met (#8198,1:3000), anti-cleaved PARP (#9541,1:1000) and anti-cleaved caspase-7 (#8438,1:1000) (Cell Signaling Technology); anti-GFP mouse (#sc-9996,1:3000), anti-ubiquitin (P4D1, #sc-8017,1:1000), anti-VPS4A (#sc-393428,1:1000), anti-VPS4B (#sc-377162,1:1000) and anti-Na/K ATPase (#sc-21712,1:1000) (Santa Cruz), anti-His (#2010762A, Takara,1:5000), anti-α-tubulin (t5168,Sigma-Aldrich,1;5000). The anti-EGFP rabbit (generated in our laboratory). Arl4A (1:3000) antibodies were previously described (Lin et al., 2011). For the EGFR trafficking assay, we used an anti-extracellular region of EGFR (#ab30, Abcam) at a concentration of 18 µg/ml. For IF, dilutions of anti-Arl4A (1:200), anti-CD63 (#GTX28219, GeneTex,1:500), anti-Myc (#2276S,1:500), anti-Lamp1 (#sc-20011,1:500) and anti-EGFR (#4267,1:500) primary antibodies and Alexa Fluor 488/594 anti-rabbit and mouse IgG secondary antibodies (A-11012, A-11001 and A-11032, Invitrogen, 1:1000) were us. For western blot, the secondary antibodies used were goat horseradish peroxidase (HRP)-conjugated anti-rabbit/mouse IgG (NA934V/NA931V, GE Healthcare, 1:5000).

Validation

The Commercial Antibodies are commercially availabl and were commercially validated.
anti-VPS36 (#PA5-60561) - validated in section of human testis for IHC and for western blot by manufacturer.
anti-HA (# MMS-101R)- validated by manufacturer with positive controls.
anti-LexA (Covance)- validated by manufacturer with positive controls.
anti-Myc (#2276S)- validated by manufacturer with positive controls.
anti-mCherry (#43590)- validated by manufacturer with positive controls.
anti-EGFR (#4267) - validated in various cell lines for ICC/IF, human lung carcinoma tissue for IHC and for western blot by manufacturer.
anti-p-EGFR (#3777) - validated in various cell lines for ICC/IF, HCC827 xenograft tissue for IHC and for western blot by manufacturer.
anti-ERK1/2 (#9107) - validated for western blot by manufacturer.
anti-p-ERK1/2 (#4370) - validated in various cell lines for ICC/IF, human breast carcinoma tissue for IHC and for western blot by manufacturer.
anti-c-Met (#8198) - validated in various cell lines for ICC/IF, human colon adenocarcinoma tissue for IHC
and for western blot by manufacturer.
anti-cleaved PARP (#9541) - validated for western blot by manufacturer.
anti-cleaved caspase-7 (#8438) - validated in various cell lines for ICC/IF, for western blot and flow cytometry by manufacturer.
anti-GFP mouse (#sc-9996)- validated by manufacturer with positive controls.
anti-ubiquitin (P4D1, #sc-8017) - validated in various cell lines for ICC/IF, human duodenum tissue for IHC and for western blot by manufacturer.
anti-VPS4A (#sc-3934280) - validated in various cell lines for ICC/IF and for western blot by manufacturer.
anti-VPS4B (#sc-377162) - validated in human uterine cervix tissue for IHC and for western blot by manufacturer.
and anti-Na/K ATPase (#sc-21712) - validated in various cell lines for ICC/IF and for western blot by manufacturer.
anti-His (#2010762A, Takara) - validated by manufacturer with positive controls.
anti-α-tubulin (Sigma-Aldrich) - validated in various cell lines for ICC/IF and for western blot by manufacturer.
EGFR (#ab30, Abcam) - validated in various cell lines for ICC/IF, section of frozen normal human placenta for IHC and flow cytometry by manufacturer.
anti-CD63 (#GTX28219, GeneTex) - validated in various cell lines for ICC/IF by manufacturer.
The anti-EGFP rabbit and Arl4A antibodies-validated by ourselves with positive controls previously described (Lin et al., 2011).
Alexa Fluor 488/594 anti-rabbit and mouse IgG secondary antibodies (A-11012, A-11001 and A-11032, Invitrogen) - validated in various cell lines and primary antibodies for ICC/IF by manufacturer.
goat horseradish peroxidase (HRP)-conjugated anti-rabbit/mouse IgG (NA934V/NA931V, GE Healthcare)- validated for western blot by manufacturer.

Eukaryotic cell lines

Policy information about cell lines and Sex and Gender in Research

Cell line source(s)

The A549 cell (BCRC Number: 60074), HeLa cell (BCRC Number: 60005), C33-A (BCRC Number: 60554) and 293t (BCRC Number: 60019) cell were purchased from Food Industry Research and Development Institute (Hsinchu, Taiwan). The PE089 was characterized as harboring an EGFR exon 19 deletion and derived from a Taiwanese female patient with adenocarcinoma of the lung (courtesy of Professor Ko-Jiunn Liu, National Health Research Institutes, Tainan, Taiwan). The H1975 cell line was provided by Professor Li-Chung Hsu (Institute of Molecular Medicine, National Taiwan University, Taipei, Taiwan). The cell line was obtained from the American Type Culture Collection (ATCC).

Authentication

The cells from Food Industry Research and Development Institute were authenticated by Bioresource Collection and Research Center (BCRC)
HeLa cell (Epithelial)
STR-PCR profile: D7S820 ： 8, 12 CSF1PO ： 9, 10 TH01 ： 7 D13S317 ： 12, 14 D16S539 ： 9, 10 vWA ： 16, 18 TPOX ： 8, 12
Amelogenin ： X D5S818 ： 11, 12
Karyology: Aneuploid; HeLa Markers: 1 copy of M1 (t(1q3q)); 1 copy of M2 (t(3p5q)); 4-5 copies of M3 (i(5p)); 2 copies of M4 (der(19)t(13;19)(q14;p13))

C33-A cell (Epithelial)
STR-PCR profile : D7S820 ： 10 CSF1PO ： 12 TH01 ： 7, 8 D13S317 ： 13 D16S539 ： 13, 14 vWA ： 18, 20 TPOX ： 9

	Amelogenin：X D5S818：11, 12)<br><br>A549 cell (Epithelial)<br>STR-PCR profile : D7S820：8, 11 CSF1PO：10, 12 TH01：8, 9.3 D13S317：11 D16S539：11, 12 vWA：14 TPOX：8, 11<br>Amelogenin：X, Y D5S818：11<br><br>293t cell (Epithelial)<br>STR-PCR profile:D7S820：11, 12 CSF1PO：12 TH01：7, 9.3 D13S317：12, 14 D16S539：9, 13 vWA：16, 19 TPOX：11<br>Amelogenin：X D5S818：8<br>Karyology: Hypotriploid; modal number = 64<br><br>The cells from the American Type Culture Collection (ATCC)<br>H1975 cell (Epithelial)<br>STR-PCR profile: D3S1358: 14,15 : TH01: 7 : D21S11: 28 : D18S51: 13 : Penta_E: 12,16 : D5S818: 11,12 : D13S317: 10,13 : D7S820: 8,11 : D16S539: 9,12 : CSF1PO: 12 :Penta_D: 12,13 :Amelogenin: X :vWA: 18 :D8S1179: 13,16 :TPOX: 8,11 :FGA: 21,24 :D19S433: 15,15.2: D2S1338: 17<br><br>The cell lines were authenticated by BCRC (HeLa, C33-A, A549 and 293t cells) and ATCC (H1975 cells). The PE089 cell line used in this study were not autherntciated. However, the PE089 was characterized as harboring an EGFR exon 19 deletion and derived from a Taiwanese female patient with adenocarcinoma of the lung (courtesy of Professor Ko-Jiunn Liu, National Health Research Institutes, Tainan, Taiwan).
Mycoplasma contamination	The cells were certified not contaminated by Bioresource Collection and Research Center (BCRC) and tested for mycoplasma contamination subsequently by PCR assay
Commonly misidentified lines<br>(See ICLAC register)	No commonly misidentified cell lines were used in the study

Animals and other research organisms

Policy information about studies involving animals; ARRIVE guidelines recommended for reporting animal research, and Sex and Gender in Research

Laboratory animals	The Arl4atm1a(EUCOMM)Hmgu ES cells were purchased from European Mouse Mutant Cell Repository (EuMMCR). The Arl4A knockout mice were generated by National Taiwan University College of Medicine Laboratory Animal Center. In this study, we adopted aged 6-7 month C57BL/6N Arl4a KO and WT mice. Male and female mice were used for experiments. In this study, we adopted aged 6-7 months C57BL/6N Arl4a KO and WT mice. Male and female mice were used for the experiments. All mice were kept in a specific pathogen free (SPF) room at temperature (22±2℃), and humidity (55±10%) with dark/light cycle (12/12h), and received food and water provided ad libitum.
Wild animals	The study did not use wild animals.
Reporting on sex	Male and female mice were used for experiments.
Field-collected samples	The study did not involve field-collected samples.
Ethics oversight	Our research complies with all relevant ethical regulations. The animal experiments were performed in accordance with National Taiwan University Institutional Animal Care and Use Committee and National Taiwan University College of Medicine Laboratory Animal Center.

Note that full information on the approval of the study protocol must also be provided in the manuscript.

Plants

Seed stocks	*Report on the source of all seed stocks or other plant material used. If applicable, state the seed stock centre and catalogue number. If plant specimens were collected from the field, describe the collection location, date and sampling procedures.*
Novel plant genotypes	*Describe the methods by which all novel plant genotypes were produced. This includes those generated by transgenic approaches, gene editing, chemical/radiation-based mutagenesis and hybridization. For transgenic lines, describe the transformation method, the number of independent lines analyzed and the generation upon which experiments were performed. For gene-edited lines, describe the editor used, the endogenous sequence targeted for editing, the targeting guide RNA sequence (if applicable) and how the editor was applied.*
Authentication	*Describe any authentication procedures for each seed stock used or novel genotype generated. Describe any experiments used to assess the effect of a mutation and, where applicable, how potential secondary effects (e.g. second site T-DNA insertions, mosiacism, off-target gene editing) were examined.*

Flow Cytometry

Plots

Confirm that:

- [x] The axis labels state the marker and fluorochrome used (e.g. CD4-FITC).
- [x] The axis scales are clearly visible. Include numbers along axes only for bottom left plot of group (a 'group' is an analysis of identical markers).
- [x] All plots are contour plots with outliers or pseudocolor plots.
- [x] A numerical value for number of cells or percentage (with statistics) is provided.

Methodology

Sample preparation	Cells were stained with an annexin V-FITC apoptosis detection kit (Invitrogen), and apoptotic cells were identified and quantified by flow cytometry according to the manufacturer's instructions. In brief, after depletion of Arl4A, the cells were washed with PBS and collected via trypsin-EDTA solution (Invitrogen). The cell suspensions were then centrifuged at 1000 rpm for 5 min to remove the trypsin-EDTA solution. Then, the cells were resuspended and incubated with propidium iodide (PI), annexin V-FITC, and annexin V binding buffer for 15 min at room temperature. The stained cells were analyzed via flow cytometry with a Muse® Cell Analyzer (luminex)
Instrument	Muse® Cell Analyzer
Software	Muse® Software
Cell population abundance	cell sorting not employed
Gating strategy	We used the Muse Cell Analyzer Annexin V & Dead Cell Assay program to study cell apoptosis. The Muse® assay-specific software program modules include sample graphs to make population gating setup foolproof. Positivity threshold for each sample was defined on the basis of C33-A siControl sample. Using the siControl sample cell size index, debris was removed by gating on the main cell population. Identical positivity threshold was applied to all samples.

- [ ] Tick this box to confirm that a figure exemplifying the gating strategy is provided in the Supplementary Information.
